# Constitutive regulation of mitochondrial morphology by Aurora A kinase depends on a predicted cryptic targeting sequence at the N-terminus

**DOI:** 10.1098/rsob.170272

**Published:** 2018-06-13

**Authors:** Rhys Grant, Ahmed Abdelbaki, Alessia Bertoldi, Maria P. Gavilan, Jörg Mansfeld, David M. Glover, Catherine Lindon

**Affiliations:** 1 Department of Genetics, University of Cambridge, Downing Street, Cambridge CB2 3EH, UK; 2 Department of Pharmacology, University of Cambridge, Tennis Court Road, Cambridge CB2 1PD, UK; 3 Cell Cycle, Biotechnology Center, Technische Universität Dresden, 01307 Dresden, Germany

**Keywords:** AURKA, mitochondria, mitochondrial dynamics

## Abstract

Aurora A kinase (AURKA) is a major regulator of mitosis and an important driver of cancer progression. The roles of AURKA outside of mitosis, and how these might contribute to cancer progression, are not well understood. Here, we show that a fraction of cytoplasmic AURKA is associated with mitochondria, co-fractionating in cell extracts and interacting with mitochondrial proteins by reciprocal co-immunoprecipitation. We have also found that the dynamics of the mitochondrial network are sensitive to AURKA inhibition, depletion or overexpression. This can account for the different mitochondrial morphologies observed in RPE-1 and U2OS cell lines, which show very different levels of expression of AURKA. We identify the mitochondrial fraction of AURKA as influencing mitochondrial morphology, because an N-terminally truncated version of the kinase that does not localize to mitochondria does not affect the mitochondrial network. We identify a cryptic mitochondrial targeting sequence in the AURKA N-terminus and discuss how alternative conformations of the protein may influence its cytoplasmic fate.

## 1. Introduction

Aurora A kinase (AURKA) was discovered as a mitotic kinase with a key role in bipolar spindle formation [1]. Its prominent localization to the microtubule spindle in mitosis is mediated through interaction of its kinase domain with the microtubule-associated protein TPX2 [2], an interaction that also activates the kinase activity of AURKA through stabilization of the active conformation of the T-loop [3]. Further studies have revealed the existence of alternative pathways to activation of AURKA [4–6] and an extensive array of cellular functions regulated by AURKA, in interphase as well as mitosis (reviewed in [7]). One such novel function that has been described for AURKA is the promotion of mitochondrial fission in preparation for mitosis [8].

Mitochondria form a highly dynamic network of interconnected tubules that undergo constant cycles of fission and fusion. These cycles are an essential feature of cellular homeostasis, thought to maintain a healthy mitochondrial population by allowing segregation of damaged mitochondria into the mitophagy pathway [9,10]. It is also proposed that fission/fusion cycles regulate the metabolic output of the cell, because a more interconnected network provides more efficient oxidative metabolism, and that mitochondrial morphology responds to metabolic cues. Mitochondrial morphology depends on the activity of GTPases dynamin-related protein 1 (Drp1) for fission and mitofusins (MFN1, MFN2) for fusion of the outer mitochondrial membrane (OMM) [11,12].

Mitochondrial fission also occurs prior to cell division in mammalian cells. Fragmentation of organelle networks (mitochondria, Golgi, ER) is a common feature of cell division, thought to improve the probability that organelles segregate equally between daughter cells [13,14] and to enable regulated segregation of aged mitochondria during stem cell-like divisions [15]. Mitochondrial fragmentation in mitosis may also be required for efficient microtubule-dependent transport of mitochondria towards the cell periphery and away from the cell division plane [16]. Indeed, blocking mitochondrial fission or transport gives rise to cytokinesis failure and aneuploidy [11,17,18], an effect that can be rescued by disrupting mitochondrial fusion [19]. Previous studies have described a pathway leading to increased mitochondrial fission at mitotic entry via Cdk1-dependent activation of Drp1 at the OMM [13]. The mitotic kinase AURKA has been proposed to regulate these events through phosphorylation of RalA to promote RalA-dependent OMM recruitment of both Drp1 and RalBP1–cyclinB–Cdk1 complex [8].

Fragmented mitochondrial networks are a characteristic of cancer cells thought to contribute to the metabolic changes that accompany tumorigenesis, and have been shown to contribute to tumour progression and metastasis [20–22]. Overexpressed AURKA (located on the 20q amplicon) is also strongly associated with cancer [23,24]. Given a large number of mitochondrial hits we identified in a search for AURKA interactors, we decided to investigate further the role of AURKA in influencing the fragmentation state of the mitochondrial network in human cell lines. We report here that the mitochondrial network is sensitive to AURKA activity at all phases of the cell cycle, and that this sensitivity contributes to divergent mitochondrial morphology between two cell lines (one transformed and the other non-transformed) expressing different levels of AURKA. Furthermore, we identify an interphase subpopulation of AURKA associated with mitochondria, both by immunofluorescence and fractionation assays. This association depends on the AURKA N-terminal region, which contains a cryptic mitochondrial targeting sequence.

## 2. Results and discussion

In using proteomic approaches to identify co-purifying proteins in AURKA-GFP pulldowns from human U2OS cells, we identified large numbers of mitochondrial proteins (electronic supplementary material, figure S1A). We confirmed these potential interactions by reciprocal pulldowns of several GFP-tagged mitochondrial markers, including the OMM translocase complex components TOMM20 and TOMM70 and the inner mitochondrial membrane component Prohibitin (PHB), which identified endogenous AURKA as a partner protein in cell extracts (electronic supplementary material, figure S1B). Repeated experiments suggested that the quantity of AURKA in pulldowns of mitochondrial proteins was at least 10-fold less than that found in pulldowns of TPX2, a major interactor of AURKA in mitotic cells [2].

**Figure 1.**
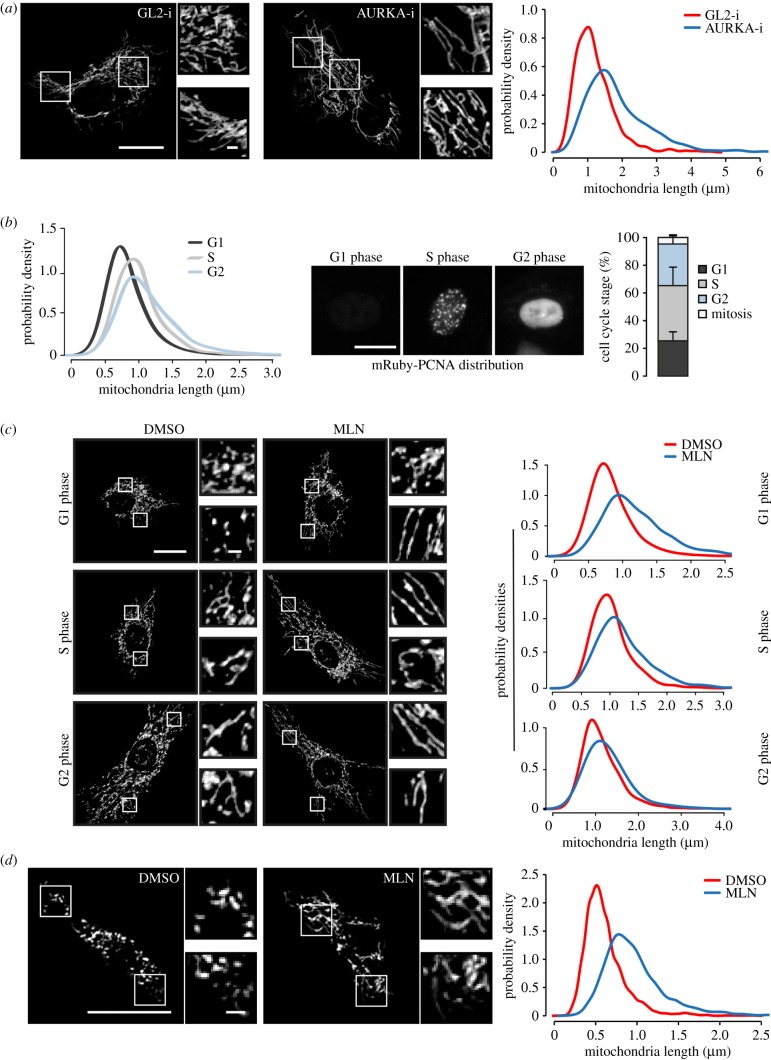
AURKA is a constitutive regulator of the mitochondrial network. (*a*) RPE-1 cells were treated with control (GL2-i) or AURKA siRNA (AURKA-i) for 48 h and mitochondria imaged in live cells using MitoTracker^TM^. Areas of cytoplasm marked by white squares are shown enlarged twofold in panels to the side of each image. Mitochondria were analysed for tubular fragment length as described in Material and methods, with raw measurements shown as probability density plots. *p* < 0.001 for the maximum deviation *D* = 0.40 (K-S test). (*b,c*) RPE-1-mRuby-PCNA cells were stained with Mito-ID^®^ green and imaged after treatment with 100 nM MLN8237 (MLN) or vehicle control (DMSO) for 3 h. (*b*) Tubular mitochondrial lengths are plotted as probability density curves (left-hand panel) for cells assigned to G1, S or G2 phase according to localization of mRuby-PCNA (middle panel), with cell cycle distribution summarized in the plot shown in the right-hand panel. (*c*) Probability density plots showing increased mitochondria length in all phases of the cell cycle after MLN treatment. G1: *p* < 0.001, *D* = 0.39; S: *p* < 0.001, *D* = 0.23; G2: *p* < 0.001, *D* = 0.13 (K-S test). (*d*) *Drosophila* D.mel-2 cells were treated with MLN for 3 h and processed as in (*a*). *p* < 0.001, *D* = 0.53 (K-S test). For panels (*b–d*): raw measurements are pooled from three statistically reproducible experimental repeats. For panels (*a–d*): scale bars, 10 µm in main panels, 1 µm in magnification panels.

Given the previous study suggesting that mitochondrial fission was controlled by a cytoplasmic pool of AURKA [8], we investigated the relevance of our own finding—of the association of AURKA with mitochondrial proteins—to mitochondrial morphology. First, we tested whether depletion of endogenous AURKA, using siRNA-mediated knockdown (AURKA-i), would affect mitochondrial morphology in the immortalized retinal pigment epithelial line hTERT-RPE-1 (RPE-1). We found that AURKA-i resulted in markedly reduced fragmentation of the mitochondrial network, observable as a more interconnected network and quantifiable as a change in length distribution of individual mitochondria (figure 1*a*; electronic supplementary material, figure S2A, B). To confirm the specificity of this effect, we treated RPE-1 cells, expressing an endogenous mRuby-PCNA marker to distinguish cell cycle phases [25], with the small-molecule-specific inhibitor of AURKA, MLN8237. We found that the length of mitochondria varied with cell cycle phase as expected [13,26], being shortest in G1 phase (figure 1*b*), but that AURKA inhibition led to elongation of mitochondria in all cell cycle phases (figure 1*c*). Therefore, we conclude that AURKA can regulate mitochondrial organization throughout the cell cycle. To determine whether this response of mitochondria to AURKA is a conserved role of the kinase, we treated cultured *Drosophila melanogaster* D.mel-2 cells with MLN8237 (figure 1*d*). The increase in mitochondrial connections and length following treatment suggest that this role for AURKA is conserved in metazoans.

**Figure 2.**
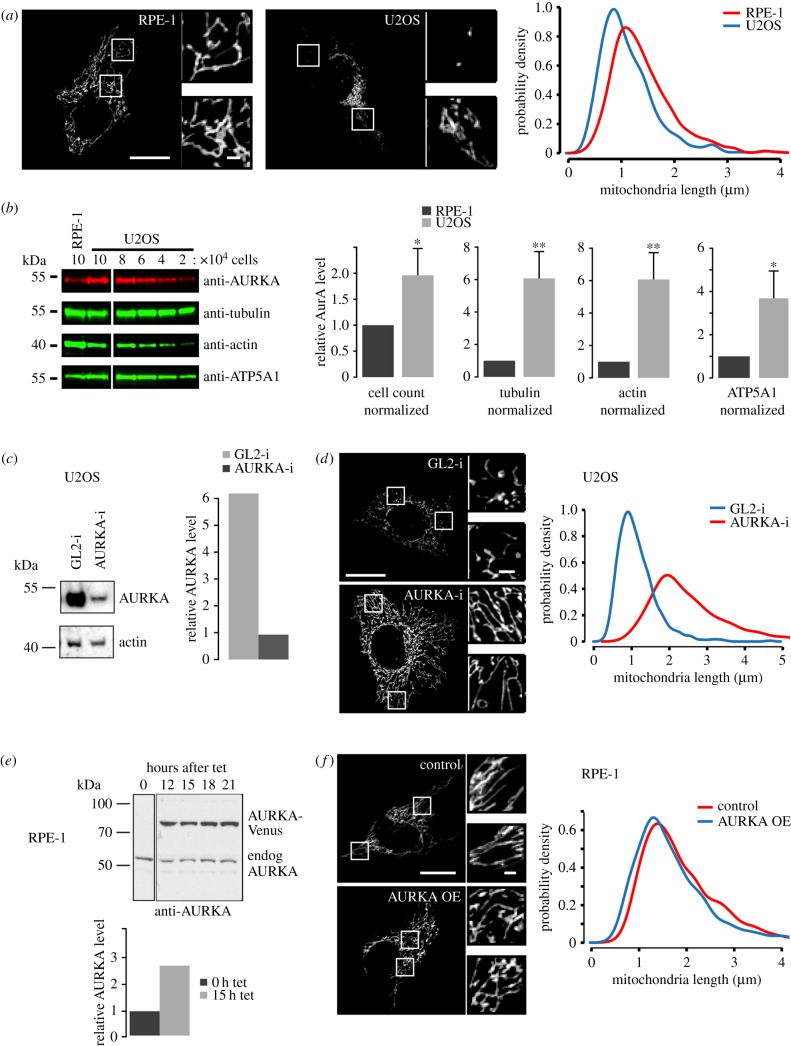
AURKA levels influence mitochondrial morphology in RPE-1 and U2OS cells. (*a*) RPE-1 and U2OS cells were treated with MitoTracker^TM^ and imaged under identical conditions. Mitochondrial lengths were measured and plotted as in figure 1. *p* < 0.001 for the maximum deviation *D* = 0.37 (K-S test) from two repeats. (*b*) Equal numbers of U2OS and RPE-1 cells were harvested for cell extracts and calculated quantities loaded onto gels to be examined by quantitative immunoblot. Bar charts show AURKA levels quantified and normalized against number of cells, or against different loading markers. Statistical confidence is indicated as *p < 0.01; **p < 0.001 (Student's *t*-test), *n* = 3 repeats. (*c,d*) U2OS cells were treated with control (GL2-i) or AURKA siRNA (AURK-i) for 48 h and then either processed for quantitative immunoblotting of AURKA levels (*c*) or stained with MitoTracker^TM^ (*d*). Example images show insets indicated by white boxes magnified twofold. Mitochondrial tubular length measurements are plotted as probability density curves. *p* < 0.001, *D* = 0.73 (K-S test). (*e,f*) RPE-1-AURKA-Venus cells were induced for AURKA-Venus expression (AURKA OE) or not (control) with addition of tet for 18 h. Cells were either processed for quantitative immunoblotting of AURKA levels (*e*) or stained with MitoTracker^TM^ for mitochondrial tubular length measurements (*f*), *p* < 0.01, *D* = 0.08 (K-S test). tet, tetracycline; endog, endogenous; OE, overexpression. For panels (*a,d,f*): scale bars, 10 µm in main panels, 1 µm in magnification panels.

We noticed that cancer cell lines used in our laboratory (e.g. U2OS) had mitochondrial networks in a more fragmented state than non-transformed RPE-1 cells (figure 2*a*; see also electronic supplementary material, figure S2). Given our finding that AURKA influences mitochondrial morphology, and the well-documented overexpression of AURKA in cancer cells, we tested whether AURKA expression levels might contribute to differences in mitochondrial morphology observed between RPE-1 and U2OS cells. We determined relative expression levels in extracts prepared from known numbers of cells in asynchronous populations, normalized against a panel of cellular proteins (figure 2*b*). This revealed that U2OS cells contain twofold higher levels of AURKA protein normalized against cell number, and fourfold to sixfold more when normalized against the levels of tubulin, actin or ATP5A1. Therefore, higher AURKA levels correlate with a more fragmented mitochondrial network. Next, we tested whether manipulating AURKA levels would reproduce observed patterns of mitochondrial organization. We used partial siRNA-mediated knockdown to achieve sixfold reduction of AURKA levels in U2OS cells, and found a corresponding lengthening of mitochondria under these conditions (figure 2*c,d*). Connectivity of the network was also greater when AURKA levels were reduced. Conversely, we found that tetracycline-induced overexpression of a stable AURKA-Venus transgene in RPE-1 cells resulted in a small but significant decrease in the length of mitochondria (figure 2*e,f*). In further experiments, we found that transient overexpression of AURKA-Venus in another untransformed line, breast epithelial MCF10A cells, also led to increased fragmentation of the mitochondrial network in cells overexpressing AURKA (electronic supplementary material, figure S2C). Finally, MLN8237 treatment of HCC1143 cells, a breast cancer line characterized by a highly fragmented mitochondrial network, caused an increase in mitochondria length (electronic supplementary material, figure S2D). Therefore we conclude that the different fragmentation state of the mitochondrial network in different cell lines is influenced by AURKA expression.

**Figure 3.**
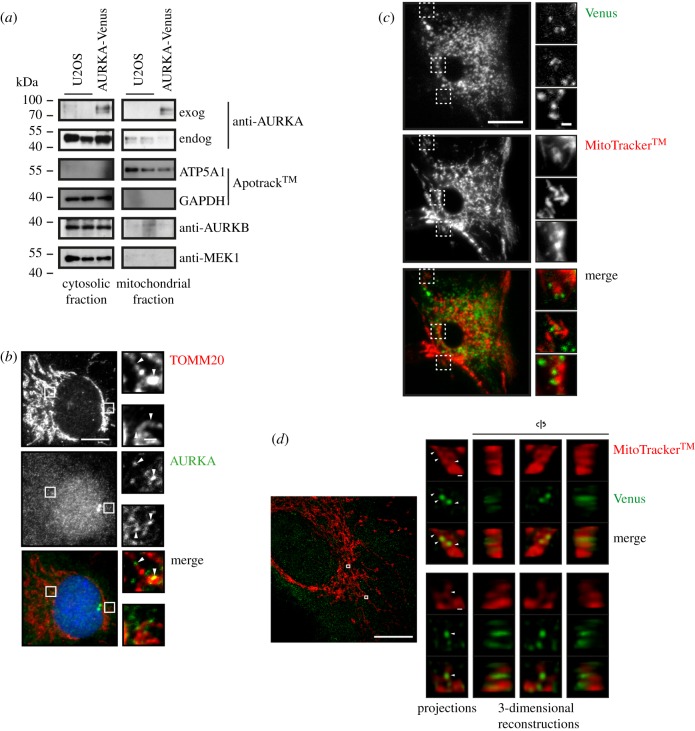
A mitochondrial fraction of AURKA. (*a*) U2OS and U2OS-AURKA-Venus cells were fractionated by serial centrifugation and probed for the presence of exogenous (exog) and endogenous (endog) AURKA, and of specific markers for cytosolic (GAPDH, MEK1) or mitochondrial (ATP5A1) fractions, by immunoblot. (*b*) Co-localization of endogenous AURKA (green) and mitochondrial marker TOMM20 (red) examined by IF in RPE-1 cells. (*c*) Live cell imaging of endogenously tagged RPE-1 mVenus-AURKA knock-in (KI) cells stained with MitoTracker^TM^ to examine co-localization. (*d*) RPE-1-AURKA-Venus cells were treated with MitoTracker^TM^ 24 h after induction of AURKA-Venus expression, then methanol-fixed, stained using GFP antibody and imaged on an OMX system. The image shown is a maximum-intensity projection of a 12 × 0.125 µm stack, with 10-fold magnification of insets. For panels (*b,c*): scale bars, 10 µm in main panels, 1 µm in magnification panels. For panel (*d*): scale bars, 10 µm in main panels, 100 nm in magnification panels.

Given the prevalence of mitochondrial hits in our AURKA interactome, and the functional relationship between AURKA and mitochondrial organization, we hypothesized that part of the cytoplasmic pool of AURKA might be associated with the mitochondrial network. In support of this, we found that both endogenous and exogenous AURKA co-purify with mitochondria in extracts fractionated by centrifugation (figure 3*a*). We also examined fixed cells for co-localization of endogenous AURKA with a mitochondrial marker, TOMM20. Immunostaining with two different antibodies against AURKA revealed punctate cytoplasmic staining in which the AURKA ‘dots’ were frequently apposed to mitochondria, often close to points of mitochondrial constriction (figure 3*b*). We then sought to confirm that these dots were AURKA-specific using siRNA to deplete endogenous AURKA. This led to the reduction of the punctate staining associated with mitochondria in accord with the downregulation of AURKA by AURKA-i treatment (electronic supplementary material, figure S3A, B). We sought to confirm our finding in live cells, by examining the cytoplasmic localization of AURKA-Venus without fixation. When AURKA-Venus was expressed exogenously from a transgene (as in figure 2*e*, also electronic supplementary material, figures S2C, S4A), levels of cytoplasmic AURKA were too high to distinguish co-localization patterns in the high cytoplasmic background. To circumvent this issue, we targeted one allele of endogenous AURKA at the C-terminus with mVenus, taking advantage of rAAV-mediated homologous recombination (electronic supplementary material, figure S3C). Endogenous mVenus-tagged AURKA was expressed to the same level as untagged endogenous AURKA, was comparably enriched in cells arrested in mitosis (electronic supplementary material, figure S3D, E) and faithfully recapitulated the known localization of untagged AURKA in the cell cycle (electronic supplementary material, figure S3F). When we examined endogenous AURKA-mVenus, we could detect a similar pattern of co-localization of cytoplasmic dots of Venus fluorescence with constrictions in mitochondria in the living cells as we had seen when staining fixed cells for endogenous AURKA (figure 3*c*). Finally, we carried out super-resolution microscopy on an OMX system and used three-dimensional reconstruction of Z-stacks to confirm that punctate GFP-labelled structures in fixed RPE-1-AURKA-Venus cells frequently coincided with constrictions in the mitochondrial network (figure 3*d*). Therefore, we concluded that a small fraction of cytoplasmic AURKA resides at mitochondria.

**Figure 4.**
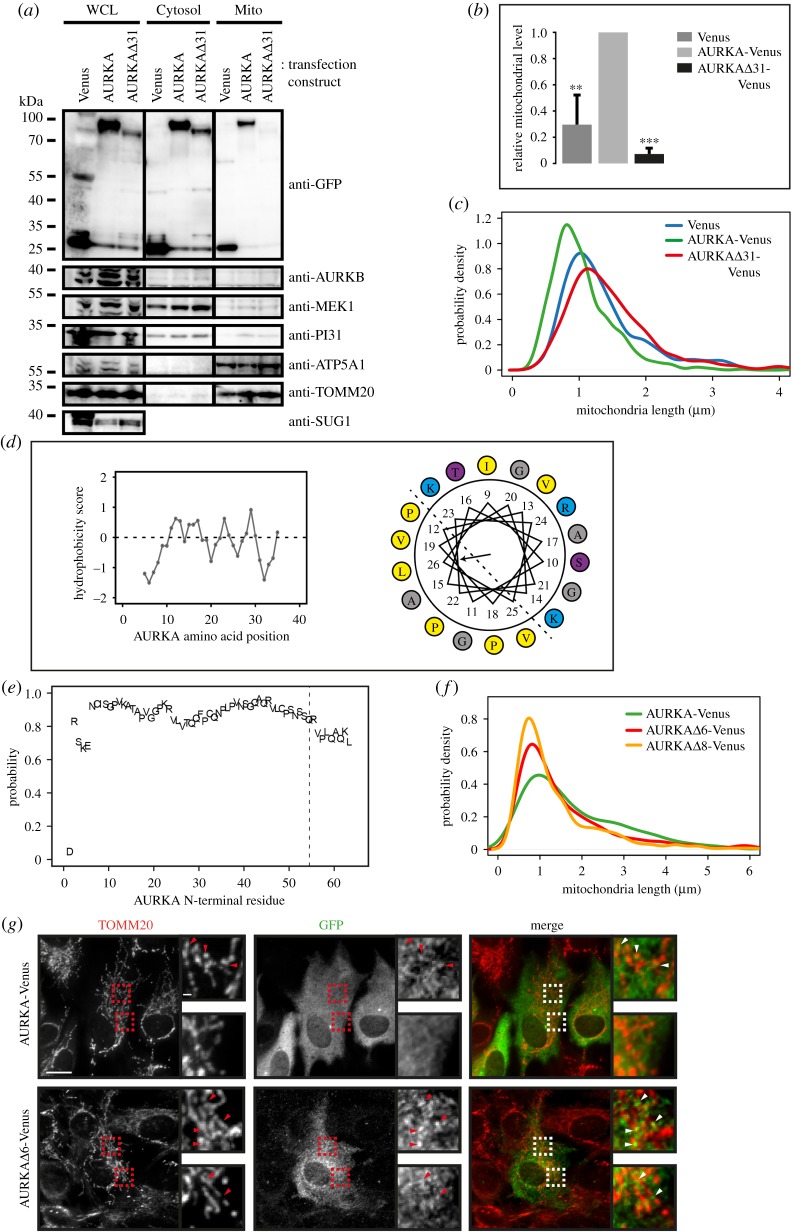
A cryptic mitochondrial targeting sequence resides in the AURKA N-terminus. (*a,b*) U2OS cells transiently transfected with AURKA-Venus (AURKA), N-terminally truncated AURKA (AURKAΔ31) or Venus alone were fractionated and probed by immunoblot (*a*) for the presence of Venus (anti-GFP) or with various markers for cytosolic (MEK1, PI31) and mitochondrial (ATP5A1, TOMM20) fractions. SUG1 was used to control for whole-cell lysate (WCL). Venus levels in the mitochondrial fraction (Mito) were quantitated and normalized against the level in WCL (*b*) in three separate experiments. **p < 0.01; ***p < 0.001 (Student's *t*-test). (*c*) N-terminally truncated AURKA (Δ31) does not cause fragmentation of the mitochondrial network. RPE-1 cells transfected with Venus-tagged AURKA, full-length and -Δ31, or Venus control, were treated with MitoTracker^TM^ and imaged under identical conditions. Mitochondrial tubular lengths are plotted as probability density curves. The two-sample K-S tests of AURKA and AURKAΔ31 populations give *p* < 0.001 for maximum deviation *D* = 0.34, from two repeats. (*d*) Likely amphipathic helix in the AURKA N-terminus revealed by Kyte–Doolittle plot (left-hand panel) and Helical wheel projection (right-hand panel) [30]. Yellow, hydrophobic; blue and purple, hydrophilic; grey, neutral. (*e*) MitoProt prediction of mitochondrial targeting probability [31] for *in silico* sequential N-terminal truncations of AURKA. (*f,g*) RPE-1 cells were transfected with wild-type or N-terminally truncated versions of AURKA-Venus (Δ6, Δ8) and imaged 24 h later after staining with MitoTracker^TM^ for measurements of mitochondrial tubular length (*f*). By two-sample K-S tests, WT versus Δ6: *p* < 0.001, *D* = 0.13; WT versus Δ8: *p* < 0.001, *D* = 0.25. Cells were then fixed and processed for IF with GFP and TOMM20 antibodies (*g*, see also electronic supplementary material, figure S5). For panel (*g*): scale bars, 10 µm in main panels, 1 µm in magnification panels.

The interaction we identified between translocase of the outer membrane (TOM) complex components and AURKA raised the possibility that AURKA might be targeted to mitochondria through a mitochondrial targeting sequence (MTS) [27,28]. These are characterized by an amphipathic helix at the N-terminus. The known structure of AURKA [29] excludes the N-terminal region, which is generally considered to be unstructured. We tested if AURKA lacking its N-terminal 31 amino acids (AURKAΔ31) would still localize in the mitochondrial fraction in cell fractionation experiments, and we found that it did not (figure 4*a,b*). Moreover, AURKAΔ31 overexpression did not cause fragmentation of mitochondria (figure 4*c*). We concluded that the N-terminal region of AURKA contains sequences necessary for its localization with mitochondria and that this localization is required for mitochondria to fragment in response to the kinase's activity. To further test this idea, we fused an MTS onto the N-terminus of AURKAΔ31. As predicted, expression of this gene fusion restored fragmentation of mitochondria (electronic supplementary material, figure S4A, B).

These findings led us to ask whether the natural N-terminal domain of AURKA had any of the characteristics of MTSs. We found that AURKA's N-terminal region is indeed polybasic and has the periodic hydrophobicity consistent with that of an amphipathic helix (figure 4*d*). This predicted amphipathic helix is a phylogenetically conserved secondary feature, even where the primary amino acid (AA) sequence diverges (electronic supplementary material, figure S4C). When we analysed the AURKA sequence using the bioinformatic algorithms, TargetP and MitoProt [31,32], we identified a ‘cryptic’ targeting sequence from AA 7–55. That is, whereas the full-length AURKA sequence was not predicted to localize to mitochondria, *in silico* removal of the first 6 AAs from its N-terminus generated a predicted mitochondrial localization signal (probability in MitoProt, *p* > 0.95; figure 4*e*). Therefore, we tested the prediction that N-terminally clipped versions of AURKA, AURKAΔ6 and AURKAΔ8, should localize more strongly to the mitochondrial network than the full-length protein. Comparing the localization of AURKA-Venus with AURKAΔ6/Δ8-Venus in fixed cells, we found that in both RPE-1 and U2OS cells, the clipped versions showed a more prominent co-localization with mitochondria, consistent with the presence of cryptic mitochondrial targeting information in the AURKA N-terminus (figure 4*g*; electronic supplementary material, figure S5). Moreover, mitochondrial fragmentation was also enhanced in the presence of AURKAΔ6/Δ8-Venus, supporting the idea that mitochondrial targeting of AURKA promotes mitochondrial fission (figure 4*f*).

A large number of proteins are described to interact with AURKA [33]. Some of these interactions may depend upon alternative conformations of the N-terminus. Thus, for example, the so-called A-box region can mediate interactions with either the APC/C cofactor Cdh1 or with Calmodulin, in a manner that may be regulated by phosphorylation on Ser51 [5,34]. We suggest that factors affecting the folding of the N-terminal region of AURKA could determine the localization and fate of the kinase by directing alternative interactions. One form of AURKA in this conformational space would display a functional MTS to mediate its mitochondrial targeting. This conformation would be favoured by AURKAΔ6.

Is AURKA on the surface of mitochondria, as predicted by its known role in promoting Drp1-mediated fission, or on the inside, as predicted by its MTS? Studies of mitochondrial regulation by mitotic cyclin-dependent kinase (Cdk) activity point to roles for cyclinB1–Cdk1 both at the mitochondrial surface, in regulating Drp1 activity [8], and—in a different study—inside the mitochondrial matrix, in regulating the respiratory chain via Complex I phosphorylation [35]. The targeting sequence on AURKA suggests that it would be translocated into the mitochondria via the TOM complex. Indeed, we have found direct interaction with the TOMM20 and TOMM70 members of this complex and also identified ATP5A subunits and other matrix components in our proteomic survey of AURKA interactors (electronic supplementary material, figure S1A).

The ‘MAGIC’ pathway, recently described by the Rong Li laboratory, diverts aggregation-prone proteins into mitochondria by an unknown mechanism that results in their ubiquitin-independent clearance by mitochondrial proteases [36]. It is possible that AURKA is an aggregation-prone protein, given the unstructured nature of its extended N-terminus. Exogenously expressed protein would be more likely to be processed via this pathway than endogenous proteins, because it would be more likely to be expressed in the absence of the correct binding partners, and we certainly cannot exclude that this pathway is responsible for co-localization of AURKA with mitochondria. However, both endogenous and exogenous AURKA localize in the same way, presumably sharing the pathway that contributes to the dynamics of the mitochondrial network.

Mitochondrial fragmentation is accompanied by increased glycolysis and decreased oxidative phosphorylation, metabolic changes that are thought to play a critical role in cell fate decisions (discussed in [37]). A switch to high glycolytic flux and low oxidative metabolism is a condition for reprogramming of iPS cells, with the state of high pluripotency being characterized by a fragmented mitochondrial network. It has been reported that AURKA is upregulated in reprogramming of iPS cells, although—curiously—inhibition of AURKA appears to enhance the process [38]. Our finding that the mitochondrial network is sensitive to AURKA levels in different cell types highlights the importance of elucidating the non-mitotic roles of AURKA in order to fully understand its contributions to cell proliferation and to cancer.

## 3. Material and methods

### 3.1. Plasmids

pVenus-N1-AURKA has been previously described [39]. Deletion mutants were made via PCR using 5′ oligos as follows:

Δ31: GAGGTACCATGCCTTGTCAGAATCCATTACC

Δ6: ATGGTACCACCATGAACTGCATTTCAGGAC

Δ8: TAGGTACCACCATGATTTCAGGACCTGTTAAGG with 3′ oligo CAGTAGGATCCGACTGTTTGCTAGCTG, and insertion via Kpn1 and BamH1 sites into pVenus-N1. An MTS from yeast TOMM70 was generated by PCR of the pMito-mCherry-FRB template [40] with oligos 5′: GCCCTCGAGATGAAGAGCTTCATTAC, 3′: CGAGGTACCCTGTTGCAATTGGTTG, and inserted between Xho1 and Kpn1 sites of AURKA-Δ31-Venus to make MTS-AURKA-Δ31-Venus. pcDNA5-FRT/TO-AURKA-Venus was made from pcDNA5^TM^/FRT/TO (Thermo Fisher Scientific) with HygromycinR sequences replaced by NeomycinR.

### 3.2. Cell culture and treatments

hTERT-RPE-1 (RPE-1) cells, RPE-1 FRT/TO-derived lines [25,41] and MCF10A cells were cultured in a 50 : 50 mix of Ham's F12 : DMEM medium, U2OS cells were cultured in high-glucose DMEM and HCC1143 in RPMI-1640 (all from Thermo Fisher Scientific). Cell culture media were supplemented with fetal bovine serum (FBS) at 10% (RPE-1, U2OS, HCC1143) or 5% (MCF10A), and with penicillin–streptomycin and amphotericin B. MCF10A cell culture was additionally supplemented with 10 mg ml^−1^ insulin, 0.5 mg ml^−1^ hydrocortisone and 20 ng ml^−1^ recombinant EGF. All cells were grown in a humidified atmosphere containing 5% CO_2_ at 37°C.

RPE-1-mRuby-PCNA cells were previously described [25]. RPE-1-AURKA-Venus (Flp-In) lines were derived as polyclonal populations by pooling cells transfected with pcDNA5-FRT/TO-AURKA-Venus and Flp-recombinase (pOG44) after 12 days of selection in 500 µg ml^−1^ geneticin. Expression of AURKA-Venus is achieved by supplementing cell culture medium with 1 µg ml^−1^ tetracycline (tet; Calbiochem, San Diego, CA, USA). U2OS-AURKA-Venus cells were obtained by clonal selection with 170 µg ml^−1^ hygromycin B (Calbiochem) after transfection of U2OS tet-OFF cells with pTRE-AURKA-Venus : pCMVhygro in a ratio of 10 : 1. Expression of AURKA-Venus in these cells is achieved with extensive washing in PBS and switching to tet-free cell culture medium. To generate the AURKA-mVenus knock-in, RPE-1 FRT/TO cells were infected with recombinant adeno-associated virus particles harbouring mVenus cDNA flanked by approximately 1500 bp homologous to the *AURKA* locus as previously described [42]. To identify positive integrands, cells were treated with 10 µM DMA 12 h before single-cell sorting by flow cytometry. mVenus-positive cells were verified by fluorescence microscopy and immunoblot analysis (electronic supplementary material, figure S3C-F). D.mel-2 cells were cultured at 25°C in Express Five^®^ serum-free medium supplemented with antibiotics and 2 mM l-glutamine.

siRNA duplex targeting human AURKA and control siRNA duplex against GL2 have been previously described [43] (Sigma-Aldrich).

Transfections were carried out using the Invitrogen Neon^®^ system to electroporate cells with siRNA or plasmids, according to the manufacturer's instructions.

For mitochondrial imaging in living cells, cells were incubated in 100 nM MitoTracker^TM^ Red CMXRos (Thermo Fisher Scientific) or Mito-ID^®^ Green (Enzo) diluted in filming medium (see below) for 15 min. MitoTracker^TM^/Mito-ID^®^ were replaced with fresh filming medium prior to time lapse.

Cells were treated with 100 nM MLN8237 during time-lapse experiments, by addition of drug in a volume not less than 1/100 of the existing dish volume and gentle mixing of the medium on the dish.

### 3.3. Cell extracts, fractionation and immunoblotting

Whole-cell extracts were prepared by scraping cells directly into 2× SDS Sample Buffer with 10 mM DTT. Samples were syringed to shear DNA or sonicated and boiled at 95°C for 5 min prior to SDS-PAGE on 4–12% precast gradient gels (Invitrogen). Transfer onto Immobilon-P or Immobilon-FL membranes was carried out using the XCell IITM Blot Module according to the manufacturer's instructions. Membranes were blocked in PBS, 0.1% Tween-20, 5% dried milk, and processed for immunoblotting with the primary antibodies indicated in the figures. Secondary antibodies used were HRP-conjugated, or IRDye^®^ 680RD- or 800CW-conjugated for quantitative fluorescence measurements on an Odyssey^®^ Fc Dual-Mode Imaging System (LI-COR Biosciences). For cell fractionation experiments, cells were harvested and treated at 4°C with 40 µg ml^−1^ digitonin in 10 mM Tris–HCl (pH 7.4), 100 mM NaCl, 25 mM MgCl_2_, 1 mM Na_3_VO_4_, 1 mM NaF and EDTA-free protease inhibitor cocktail (Roche). Cells were disrupted by 10 passages through a 25-gauge needle (whole-cell extract). Cell nuclei were pelleted by centrifugation at 3000 r.p.m. for 10 min in a microfuge at 4°C. The resulting supernatant was re-centrifuged at 13 000 r.p.m. for 15 min to yield mitochondrial (pellet) and cytoplasmic (supernatant) fractions.

### 3.4. Immunofluorescence analysis

Cells were grown on 13 mm glass coverslips, synchronized and fixed using either 100% MeOH at –20°C or 4% PFA treatment at ambient temperature. Mitochondria were detected using anti-TOMM20 (Santa Cruz), AURKA using anti-AURKA (rabbit antibody Abcam ab1287 or mouse antibody BD Transduction Laboratories 610 939) and AURKA-Venus using anti-GFP (Abcam ab290). Secondary antibodies used were Alexa^®^Fluor 488-anti-rabbit and Alexa^®^Fluor 568-anti-mouse (Thermo Fisher Scientific). Cells were stained according to standard protocols in PBS buffer containing 0.1% Triton X100 and 3% BSA and mounted in ProLong^®^ Gold antifade (Thermo Fisher Scientific). Images were captured using an Axiovert 200 M fluorescence microscope (Carl Zeiss Inc.) with a 100× NA 1.4 oil objective and Coolsnap HQ2 camera (Photometrics) controlled by the MetaMorph^®^ software. Images were deconvolved using 10 iterations of the AUTOQuant X2 (Media Cybernetics) blind deconvolution algorithm and presented as maximum-intensity projections of 10 × 0.1 µm stacks using ImageJ (Molecular Devices LLC). Super-resolution images were acquired on a Deltavision Optical Microscope eXperimental (OMX) 3D-SIM System V3 and analysed using softWoRx (Applied Precision) and ImageJ Volume Viewer plugin. Intensity level, contrast and brightness of images were adjusted using Adobe Photoshop where indicated.

### 3.5. Live cell microscopy

Cells were seeded onto 8-well plastic-bottom slides (Ibidi GmbH, Martinsried, Germany) at a density of 8 × 10^4^ cm^−2^. The imaging medium was Leibowitz L-15 (Thermo Fisher Scientific) supplemented with FBS and antibiotics as described above. Expression of AURKA-Venus was induced 24 h prior to imaging, which was carried out on an Olympus CellR widefield imaging platform comprising an Olympus IX81 motorized inverted microscope, Orca CCD camera (Hamamatsu Photonics, Japan), motorized stage (Prior Scientific, Cambridge, UK) and 37°C incubation chamber (Solent Scientific, Segensworth, UK) fitted with appropriate filter sets and a 60× NA 1.42 oil objective. Images were acquired using the Olympus CellR software as 1 µm stacks and exported as 12 bit TIFF stacks for display as maximum-intensity projections in ImageJ.

### 3.6. Mitochondrial morphology analysis

MitoTracker^TM^ images were analysed using the MicroP software [44] to derive measurements of mitochondrial length. All analyses and statistical significance tests were verified through a separate manual analysis of the same images using the lengths of 30 mitochondria per cell, selected by beginning at the 12 o'clock position and moving clockwise around the nucleus. MicroP results were plotted in R as kernel density estimations to derive the presented probability density curves (see electronic supplementary material, figure S2A). Statistical significance of probability density distributions were tested using a two-sample Kolmogorov–Smirnov (K-S) test on the raw measurements as indicated in figure legends and illustrated in the electronic supplementary material, figure S2B. All results reported in this study were also significant according to the Mann–Whitney *U*-test, *p* < 0.01.

## Supplementary Material

Supplementary material

## Data accessibility

The datasets supporting this article have been uploaded as part of the electronic supplementary material.
