# Supplementary material for: Constitutive regulation of mitochondrial morphology by Aurora A kinase depends on a predicted cryptic targeting sequence at the N-terminus
Source: Open Biol. 2018 Jun 13;8(6):170272. doi: 10.1098/rsob.170272 (PMC6030116; doi:10.1098/rsob.170272)
Supplement: Supplementary material [file rsob170272supp1.pdf]

Supplementary information for

Constitutive regulation of mitochondrial morphology by Aurora A kinase depends on a predicted cryptic targeting sequence at the N-terminus

Rhys Grant, Ahmed Abdelbaki, Alessia Bertoldi, Maria Paz Gavilan, Jörg Mansfeld, David M Glover, Catherine Lindon

DOI: 10.1098/rsob.170272

Supplementary Methods (to accompany Figure S1)

Supplementary Figures S1 – S5

Supplementary References

Supplementary Methods

Proteomic identification of AURKA interactors

GFP-Trap® beads (10 µL agarose beads or 5 µL magnetic beads per 10^7 cells) were prepared by rinsing 3 times in Dilution Buffer (10 mM Tris-HCl, pH 7.5, 150 mM NaCl, 0.5 mM EDTA, 1 mM Na_3VO_4, 1 mM NaF, protease inhibitor cocktail mix (EDTA-free)). 24 h after induction of AURKA-Venus expression, RPE-1-AURKA-Venus cells, U2OS-AURKA-Venus cells and parental cells (negative controls) were harvested and lysed in 120 µL Lysis Buffer (0.5% (v/v) NP-40, 10 mM Tris-HCl, pH 7.5, 150 mM NaCl, 0.5 mM EDTA, 1 mM Na_3VO_4, 1 mM NaF, protease inhibitor cocktail mix (EDTA-free)). Lysate was diluted to a total volume of 300 µL using Dilution Buffer, was applied to the beads, and was incubated at 4 °C for 2 h on a rotating wheel. Supernatant was removed, and the beads were washed 5 x 5 mins in Dilution Buffer. Bound proteins were eluted by boiling beads in 50 µL Elution Buffer (2X NuPAGE® LDS sample buffer, 10 mM DTT, in PBS). Eluate was rapidly transferred to a clean eppendorf tube and was submitted for mass spectrometry analysis. Samples were reduced via DTT, alkylated via iodoacetamide, and then digested with trypsin at 37 °C for 16 h. Peptide mixtures were analysed by high-performance liquid chromatography tandem mass spectrometry using a nanoAcquity UPLC (Waters) system and an LTQ Orbitrap Velos hybrid ion trap mass spectrometer (Thermo Fisher Scientific). Data were converted to .mgf files using Protein Discoverer (Thermo Fisher Scientific). Raw .mgf files were submitted to the Mascot

search algorithm (Matrix Science) allowing for 2 missed cleavages, fixed modifications (Carbamidomethyl (C)) and variable modifications (Oxidation (M), Phospho (ST), Phospho (Y), Gly-Gly (K)), with a peptide tolerance of ± 25 ppm, a MS/MS tolerance of ± 0.8 Da, and peptide charges of 2+, 3+ and 4+.

To identify AURKA-specific hits, data were pooled from 18 samples prepared in three separate experiments. Hits absent from all negative controls were classed as being AURKA-specific. For non-specific hits (which included known GFP-Trap® contaminants [1]), the ratio of maximum AURKA pulldown Mascot score to maximum negative control pulldown Mascot score was calculated using data from all experiments. A cut-off ratio was set at that of our lowest scoring known AURKA interactor Nucleophosmin (1.875). Non-specific hits with a ratio below this were deemed contaminants and were removed completely from analyses. As an additional level of control, for the remaining non-specific hits, the ratio of AURKA pulldown Mascot score to negative control pulldown Mascot score was calculated for each individual experiment. The same cut-off ratio of 1.875 was applied. Any non-specific hits with a ratio below this in an individual experiment were deemed to not be significant and were excluded from analyses of that particular experiment.

Supplementary Figures

A

Gene name	Mascot score	peptides
**AURKA**	1288	60
Known interactors		
**TPX2**	144	4
**NPM1**	315	9
**PPP1R12A**	548	21
**CALM**	100	3
**ARPC3**	217	10
**EB3**	134	5
Mitochondrial hits		
**ATP5A1**	1136	44
**COX5A**	235	8
**HSPA9**	1364	52
**PHB**	726	26
**PHB2**	922	43
**PRDX3**	243	11
**SLC25A5**	442	16
**TUFM**	785	27

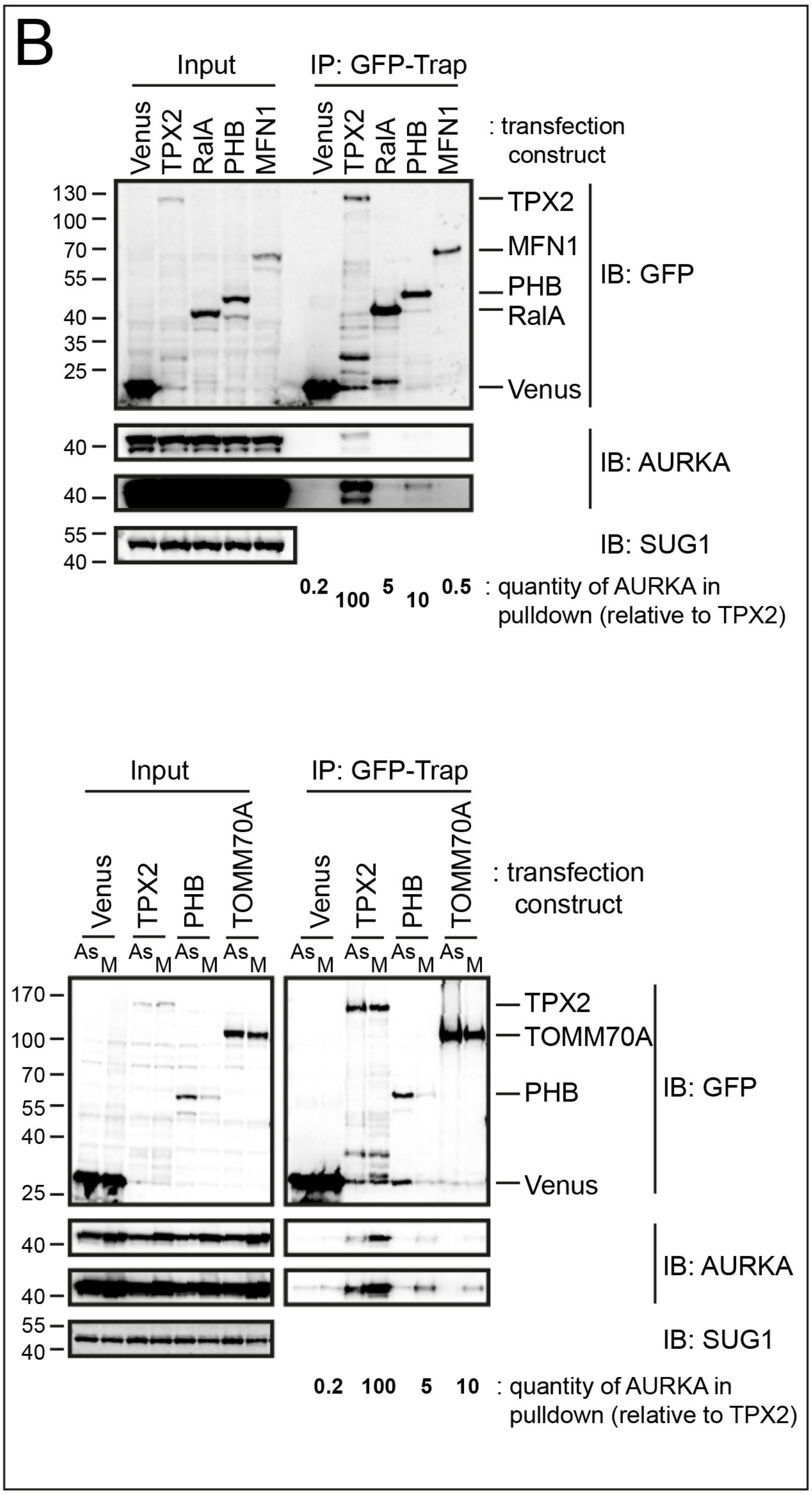

Figure S1: AURKA interacts with mitochondrial proteins

A Table of hits from mass spectrometry interrogation of AURKA-Venus pulldowns. Pulldowns were carried out using GFP-Trap® methodology, as detailed in Supplementary Methods. 'Mitochondrial hits' lists gene names with mitochondrial annotations that (1) were present in more than one replicate from at least two separate experiments (2) had maximum Mascot score in any experiment ≥ 100 and (3) showed greater than 2-fold increase in Mascot score relative to control Venus pulldowns. For comparison, the Mascot scores and corresponding peptide numbers are shown for some of the known interactors of AURKA that we found in the pulldowns. Further discussion of validity of these hits is found in Supplementary Methods section. **B** Pulldowns of endogenous AURKA by Venus-tagged candidate interactors. Quantifications of AURKA in pulldowns show ≤ 1/10 of quantity that co-purifies with TPX2 from mitotic cells. IP, immunoprecipitation; IB, immunoblot; As, asynchronous; M, mitotic.

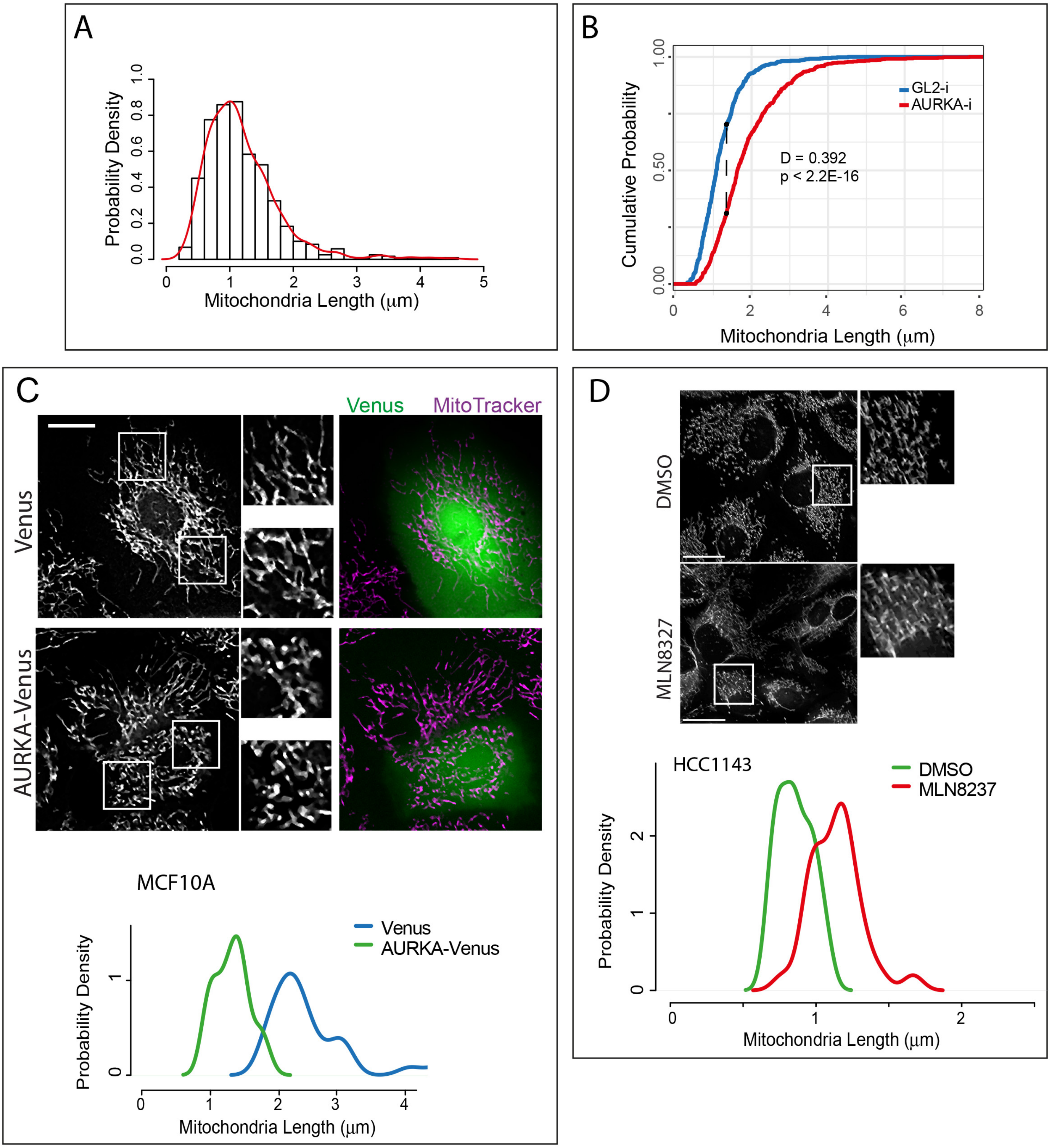

Figure S2: AURKA modulates mitochondrial morphology

A, B Supplementary information to accompany mitochondrial tubular lengths analysis in Figure 1A. **A** Probability densities from control (GL2i) samples plotted as a histogram with associated unsmoothed kernel density function, as used for all figures. **B** Two-sample cumulative probability plot according to Kolmogorov-Smirnov (K-S) analysis. **C** MCF10A cells were transiently transfected with plasmids expressing AURKA-Venus, or Venus only, and after 24 h stained with MitoTracker^{TM} for live imaging. Mitochondrial tubular length measurements are plotted as probability density curves. $p < 0.001$ for maximum deviation $D = 0.45$ (K-S test). Example images show insets indicated by white boxes magnified two-fold. **D** HCC1143 cells (with highly fragmented mitochondria) were treated with 100 nM MLN8237, or DMSO control treatment, for 2 h prior to staining with MitoTracker^{TM} for live imaging. Example images show insets indicated by white boxes magnified two-fold whilst mitochondrial tubular length measurements are plotted as probability density curves. $p < 0.001$, $D = 0.21$ (K-S test). Scale bars, 10 μM.

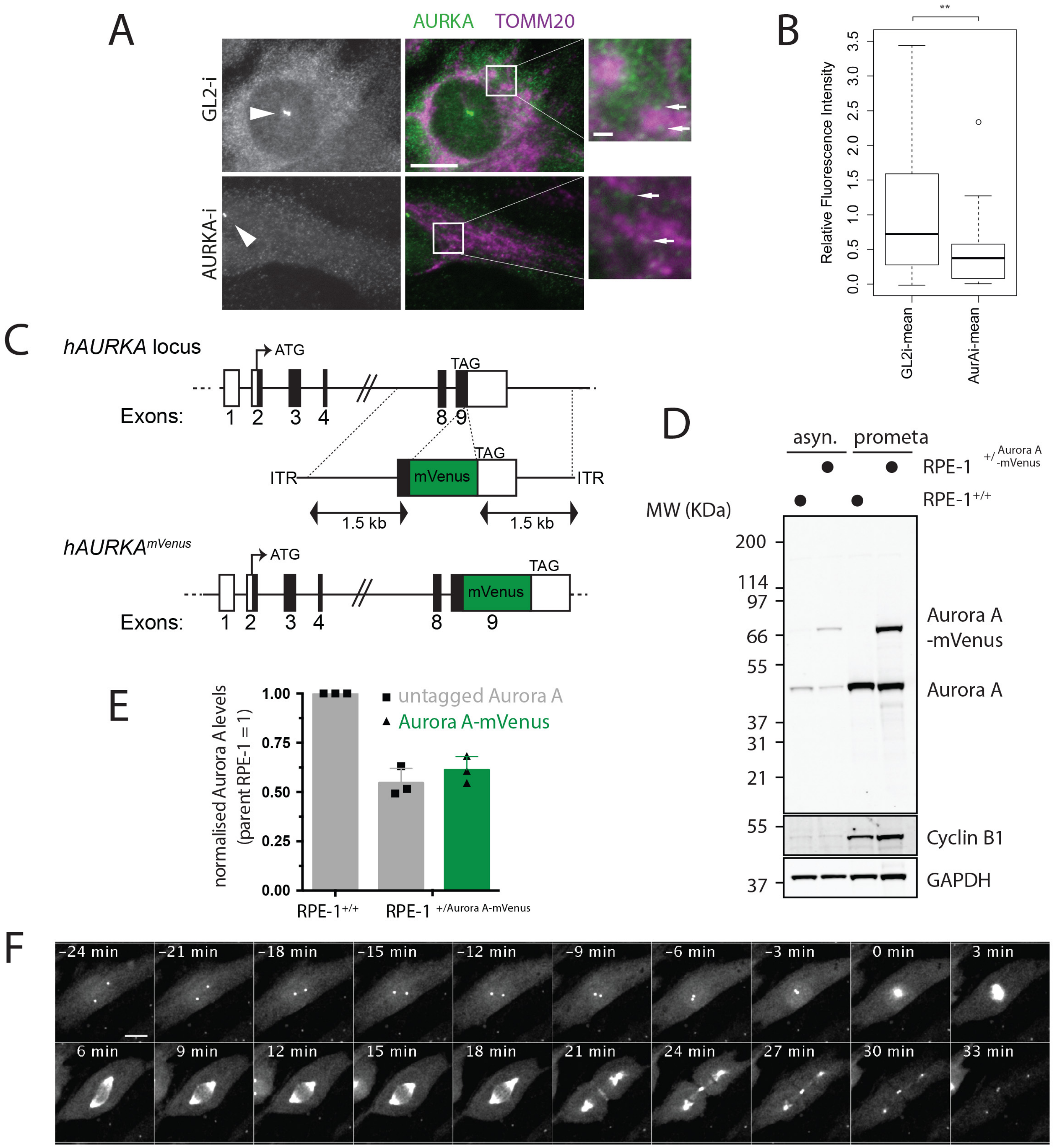

Figure S3: Detecting endogenous AURKA

A, B Indirect immunofluoresence analysis of AURKA levels in fixed cells after treatment with control (GL2-i) or AURKA siRNA. Representative cells stained for AURKA and TOMM20 are shown in **A**. Scale bars, 10 μm in main panels, 1 μm on magnification panels. Note AURKA still strongly retained at centrosomes after AURKA siRNA (arrowheads). Arrows indicate co-localization of cytoplasmic dots with mitochondrial staining, with intensity of dots reduced after AURKA-i treatment. Whole cell quantifications of AURKA levels in individual cells from one of three repeat experiments are plotted as boxplots in **B**, $p < 0.01$ (Student's t-Test). **C-F** Tagging endogenous AURKA with mVenus. **C** Strategy for C-terminal tagging of endogenous AURKA with mVenus. **D** Representative Western blot analyses of lysates from parent RPE-1 (+/+) cells and cells heterozygous for AURKA-mVenus (+/Aurora A-mVenus) that were asynchronously growing (asyn.) or were arrested in prometaphase (prometa) with 10 μM dimethylenastron (DMA) for 12 h. **E** Quantification of AURKA levels comparing the amount of untagged AURKA in the parent RPE-1 cell line with untagged and mVenus tagged AURKA in RPE-1 +/AURKA-mVenus cells. Data represent the mean and SD from three experiments. **F** Montage of time-lapse images of a representative RPE-1+/AURKA-mVenus cell across mitosis, to confirm that the tagged allele of AURKA is expressed and localizes as endogenous AURKA. Nuclear envelope breakdown, t=0. Scale bar, 10 μM.

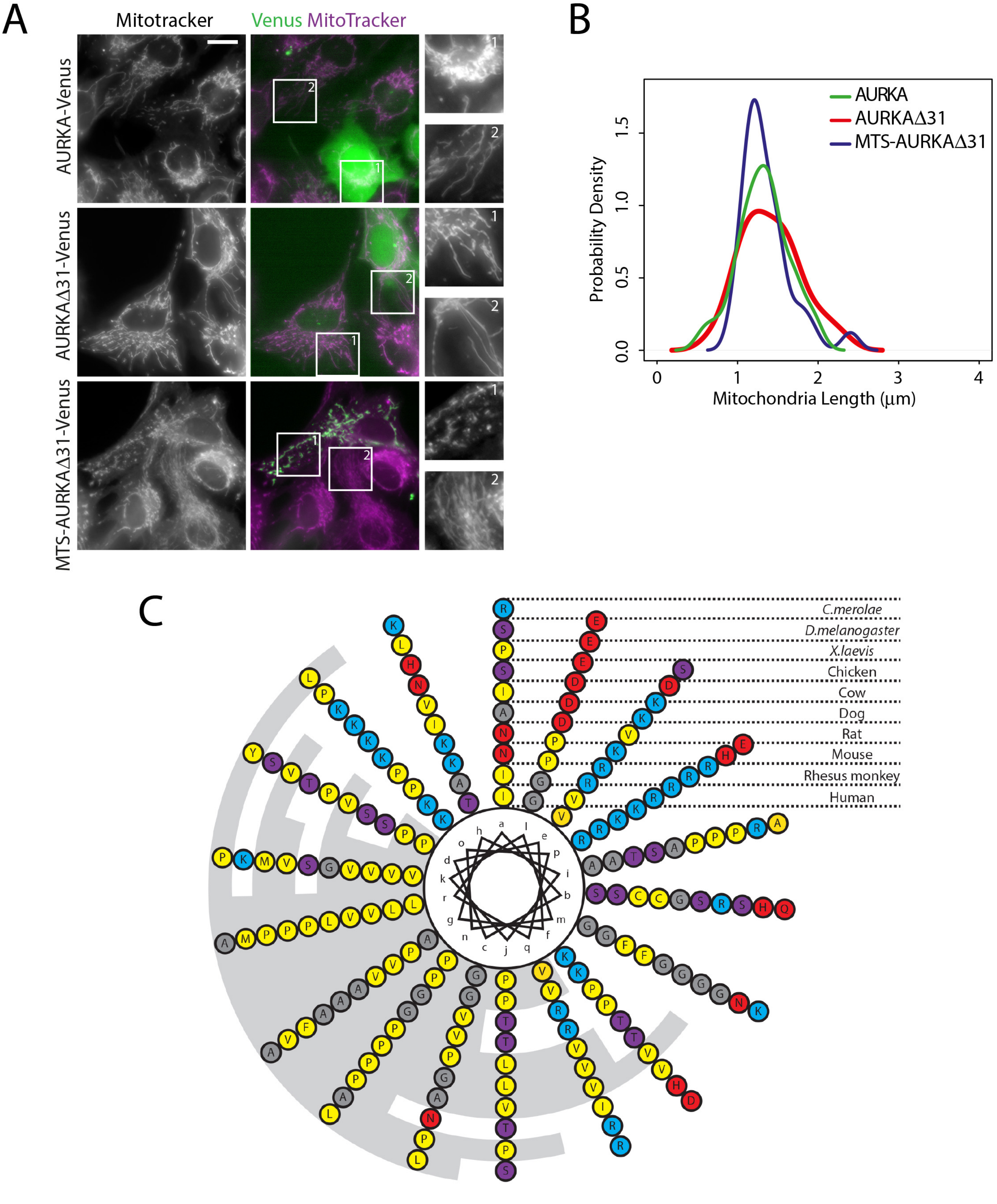

Figure S4: Mitochondrial targeting of AURKA induces fission

A, B Ectopic targeting of AURKA (Δ31) to mitochondria is sufficient to cause mitochondrial fission. RPE-1 cells were transfected with WT and Δ31 versions of AURKA-Venus, alongside a MTS-Δ31 fusion and stained with MitoTracker^{TM} after 24 h for live imaging. MTS, mitochondrial targeting sequence. **A** Representative fields of cells are shown expressing different Venus-tagged sequences. In each field, mitochondrial staining from one Venus-positive (1) and one control cell (2) are enlarged in right-hand panels. Scale bar, 10µm. **B** Mitochondrial lengths were measured and probability densities plotted. Two-sample K-S test on Δ31 and MTS-Δ31 populations gives $p < 0.001$ for maximum deviation $D = 0.20$. **C** The amphipathic helix in 1-31 of AURKA is phylogenetically conserved. Helical wheel projection of amphipathic region from Clustal Omega sequence alignment of various eukaryotic species, calculated using HELIQUEST [2, 3]. Grey box indicates the hydrophobic face. Yellow, hydrophobic; blue, hydrophilic; purple, hydroxylated; red, polar; grey, neutral.

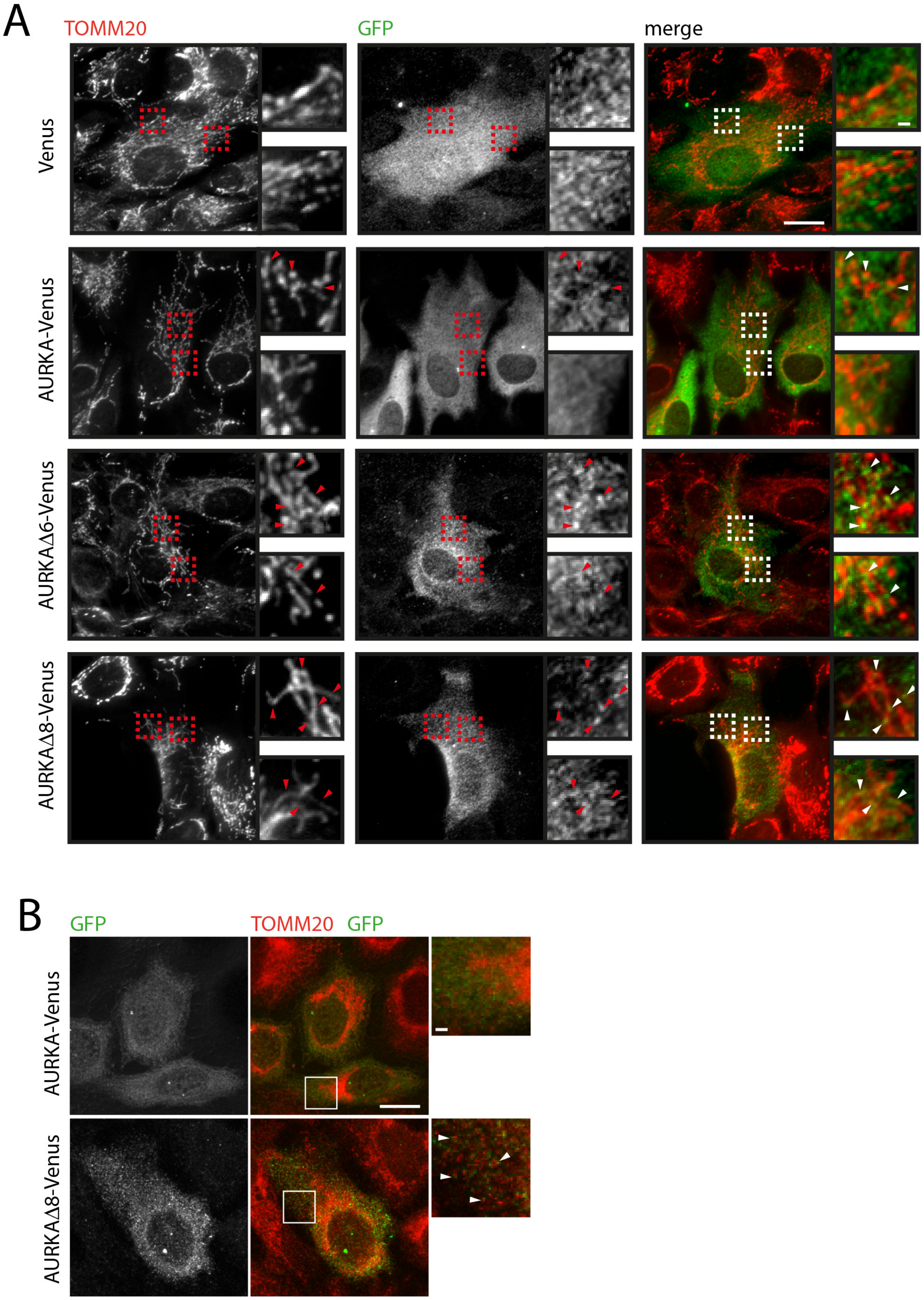

Figure S5: N-terminally clipped AURKA shows increased co-localization with mitochondria

RPE-1 cells (**A**) or U2OS cells (**B**) were transfected with wild-type or N-terminally truncated versions of AURKA-Venus (Δ6, Δ8) and after 24 h fixed and processed for IF with GFP and TOMM20 antibodies. Arrowheads indicate AURKA-Venus dots that colocalize with mitochondria. Scale bars, 10 μm in main panels, 1 μm on magnification panels.

Supplementary References

1 Trinkle-Mulcahy, L., Boulon, S., Lam, Y. W., Urcia, R., Boisvert, F. M., Vandermoere, F., Morrice, N. A., Swift, S., Rothbauer, U., Leonhardt, H.*, et al.* 2008 Identifying specific protein interaction partners using quantitative mass spectrometry and bead proteomes. *The Journal of cell biology*. **183**, 223-239. (10.1083/jcb.200805092)

2 Gautier, R., Douguet, D., Antonny, B., Drin, G. 2008 HELIQUEST: a web server to screen sequences with specific alpha-helical properties. *Bioinformatics*. **24**, 2101-2102. (10.1093/bioinformatics/btn392)

3 Sievers, F., Wilm, A., Dineen, D., Gibson, T. J., Karplus, K., Li, W., Lopez, R., McWilliam, H., Remmert, M., Soding, J.*, et al.* 2011 Fast, scalable generation of high-quality protein multiple sequence alignments using Clustal Omega. *Molecular systems biology*. **7**, 539. (10.1038/msb.2011.75)
